# Poly (Propylene Carbonate) with Extremely Alternating Structure Used as Binders for High-Loading Cathodes by Solvent-Free Method in High-Performance NCM811 Batteries

**DOI:** 10.3390/ma17225466

**Published:** 2024-11-08

**Authors:** Zhe Zhang, Jinyin Ma, Min Xiao, Shuanjin Wang, Sheng Huang, Hui Guo, Dongmei Han, Yuezhong Meng

**Affiliations:** 1 School of Chemical Engineering and Technology, Sun Yat-sen University, Zhuhai 519000, China; zhangzh569@mail2.sysu.edu.cn (Z.Z.); e1374520@u.nus.edu (J.M.); guoh37@mail.sysu.edu.cn (H.G.); 2 The Key Laboratory of Low-Carbon Chemistry & Energy Conservation of Guangdong Province/State Key Laboratory of Optoelectronic Materials and Technologies, School of Materials Science and Engineering, Sun Yat-sen University, Guangzhou 510275, China; stsxm@mail.sysu.edu.cn (M.X.); wangshj@mail.sysu.edu.cn (S.W.); huangsh47@mail.sysu.edu.cn (S.H.); 3 Institute of Chemistry, Henan Academy of Sciences, Zhengzhou 450000, China; 4 College of Chemistry, Zhengzhou University, Zhengzhou 450001, China

**Keywords:** poly (propylene carbonate), binder, solvent-free, dry electrode, lithium-ion batteries

## Abstract

The cathode affects the capacity, working voltage, and cost of lithium-ion batteries. Although the binder is a small part of the cathode material, it is particularly important to the performance of the batteries. Therefore, the design and development of polymer binders with different structures and characteristics is an important topic. In this paper, an NCM811 cathode (PPC-NCM) was prepared by a solvent-free method using poly (propylene carbonate) (PPC) as the binder, with an active substance loading of 10 mg/cm^2^. To explore the effect of the PPC binder on the electrochemical performance of the NCM811 cathode, the discharge capacity was 112.2 mAh/g with a 76.1% capacity retention after cycling more than 200 cycles at 1 C, which has a significantly better cycling performance than that of a PVDF-NCM/Li battery. The PPC/NCM/graphite full cells were also assembled to demonstrate the practical application potential of this work. It was shown that PPC as a binder can improve the cycling stability of NCM811/Li and NCM811/graphite full cells. The PPC binder used in the NCM811 cathode not only makes it extremely easy to prepare dry electrodes, but also makes it very simple to recover the electrode material by heating in the case of battery failure. This paper provides a new idea for the industrialization and development of a novel binder.

## 1. Introduction

Lithium-ion batteries (LIBs) are pivotal in powering a wide range of devices, from electronics to electric vehicles (EVs) [1,2,3,4]. LiNi_0.8_Co_0.1_Mn_0.1_O_2_ (NCM811), a Ni-rich layered cathode material known for its high specific capacity and relatively low cost, is considered one of the most promising candidates for NCM materials [5,6]. Despite its advantages, the NCM811 cathode experiences a gradual loss of capacity and significant thermal runaway during prolonged cycling [7,8]. These issues arise from adverse side-reactions at the cathode/electrolyte interface, surface structural degradation of the cathode, and poor thermal stability. Many techniques have been proposed to modify the surface properties of NCM811 to improve battery performance [9]. Techniques used to modify the surface properties of NCM811 focus on coating [10,11], doping [9,12], and surface chemistry modifications [13] to enhance battery performance. In addition to the above strategies, optimizing cathode binders to enhance the overall performance of NCM811 batteries has attracted more attention.

As is well known, the performance and longevity of these batteries are significantly influenced by the choice of binder in the cathode. Choosing the right binder can be a straightforward and efficient approach to bolstering electrode stability; they enhance mechanical strength and ensure interconnected structures, facilitating electronic and ionic transfer throughout battery cycling, and improving the batteries’ electrochemical performance [14,15,16]. Traditionally, polyvinylidene fluoride (PVDF) binders have been extensively used in lithium-ion batteries. However, it has several disadvantages. The chemical instability of PVDF causes PVDF to degrade under high voltages, leading to capacity loss over time. PVDF has relatively poor adhesion properties with NCM particles [17], which can lead to mechanical instability, especially during cycling, resulting in reduced battery performance [18,19]. PVDF is not ionically conductive, which can impede lithium-ion transport within the electrode, potentially reducing the overall battery performance. The processing of PVDF requires the use of toxic and harmful solvents like NMP (N-Methyl-**2**-pyrrolidone), which pose environmental and health hazards. To solve the mentioned problems, polymer binder with a certain Li+ conductivity was considered to prepare the electrode by a solvent-free method, which solves both the solvent pollution and the high energy loss of production. The binder for the NCM cathode should have both excellent thermal and chemical stability to prevent degradation in extreme environments such as high voltage and high temperature, which can damage the electrode structure and cause electrode side-reactions. At the same time, it should be able to form a strong bond with the active materials to effectively reduce the mass loss of the active materials or the destruction of the electrode structure during cycling.

In our previous work, we used PPC and chain extension-poly (propylene carbonate)-based polymers as binders, and we used dry electrode preparation methods to apply to dry LiFePO_4_ (LFP) electrodes, all of which demonstrated good electrochemical properties [20,21]. In this study, it was found that the processing energy consumption could be reduced by the solvent-free method [21]. Therefore, we innovatively applied the industrial PPC synthesized by our group to the NCM solvent-free system. Since the polymer can be completely decomposed under heat treatment, the material can be recycled through a simple process after the battery life expires, saving costs. Based on this, herein, PPC was used as the cathode binder, and a high-load-capacity lithium-ion battery cathode (PPC-NCM) was prepared by the solvent-free method with an active material loading of 10 mg/cm^2^. The effect of the PPC binder on the electrochemical performance of PPC-NCM was investigated. The discharge capacity was 112.2 mAh/g with a capacity retention rate of 76.1% after more than 200 cycles at 1 C, which has a significantly better cycling performance than that of a PVDF-NCM/Li battery. The PPC/NCM/graphite full battery was also assembled to demonstrate the potential of the binder for practical applications.

## 2. Materials and Methods

### 2.1. Materials

LiNi_0.8_Co_0.1_Mn_0.1_O_2_ (NCM811) from Canrd Co., Ltd. (Guangzhou, China) was used as the active material. Conductive carbon black (Super P) from Canrd Co., Ltd. (Guangzhou, China) and carbon nanotubes (CNTs) from JCNO Co., Ltd. (Nanjing, China) were used as the conductive material. Carbon coated aluminum foil was used as collector. A polyvinylidene difluoride (PVDF) binder was obtained from LIGE science Co., Ltd. (Guangzhou, China), and poly (propylene carbonate) (PPC) was purchased from Tianguan Co., Ltd. (Nanyang, China) ($\overset{—\;—}{M}$*_w_*≈ 40,000).

### 2.2. Purification of Binder

PPC was dissolved in dichloromethane, HCl was added in a ratio of 1:1 by volume with stirring, and the supernatant was poured out; this step was repeated three times. Next, deionized water was added in the same ratio and the process was repeated three times. Finally, the second layer of liquid was dropped into ethanol with stirring, the precipitate was put into an oven to dry, and then pure PPC could be obtained.

### 2.3. Preparation of Cathode

Firstly, conductive carbon Super P and CNT were mixed in a ratio of 2:8 to obtain the mixed conductive additive; then, the active material (NCM811), conductive additive, and binder were mixed and milled in a ratio of 88:10:2 and then mixed for 30 min with a high-speed mixer at 25,000 rpm. The cathode prepared by the solvent-free method was obtained by hot pressing at 170 °C and 8 MPa for 10 min to attach it to the collector. The active material loading of the cathode was about 10 mg/cm^2^.

**Figure 1:**
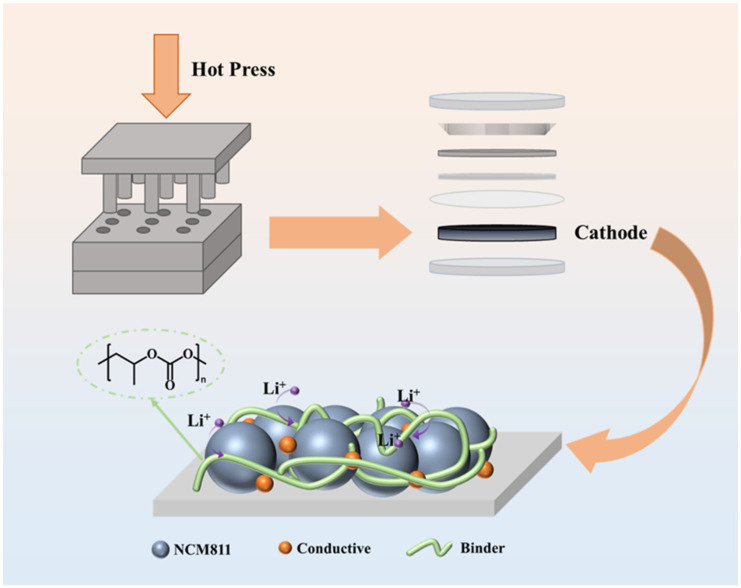
Schematic diagram of preparation of solvent-free dry NCM811 cathode with PPC binder.

We prepared solvent-free dry electrodes using NCM811 as the cathode active material and PPC as the binder. PPC, synthesized from carbon dioxide and epoxypropane, has been shown in previous studies to be in the flow dynamic phase at 150 °C [21], which is favorable for adhesion of the dry electrode and fluidity of the binder. PPC can become uniformly distributed in electrode materials and makes PPC more suitable for use in dry electrodes. Due to its rich carbonate groups, PPC promotes lithium-ion migration and exhibits good Li^+^ conductivity. Figure 1 illustrates the preparation of the solvent-free dry NCM811 cathode with a PPC binder.

### 2.4. Characterizations

The homogeneous distribution of the binder in cathode powder was tested by TGA (TG209F1 Libra, Selb, Germany) in a temperature range of 30–600 °C with a heating rate of 10 °C/min. The morphology changes of the electrode surface before and after the charge and discharge cycle were observed by SEM (SU8010, Hitachi, Tokyo, Japan). The adhesion of the binder was tested using an electronic tensile tester (CG10, Labthink LanGuang, Jinan, China). The tests consisted of two steps, compression and tension, and the whole process was carried out on a uniaxial tensile testing machine in order to apply accurate and repeatable force. Both sides of the cathode were bonded with 3M double-sided tape, and programmed compression and tension were performed to ensure uniform bonding. Through the program setting, the compression process was maintained for 60 s at 80 N and then tested at a stripping speed of 100 mm/min [20].

To evaluate the electrochemical performance of the NCM811 cathode, a 2025 coin cell was assembled in a glove box with a high-purity Ar atmosphere using a PP separator (Celgard 2500, Guangzhou, China), lithium anode, and electrolyte (Ternary universal electrolyte). Charge–discharge tests were performed at 25 °C in the range of 2.8–4.5 V at 1 C (CT2001A, Wuhan, China). Electrochemical impedance spectroscopy was determined using an electrochemical workstation (CHI604E, Shanghai, China) with a frequency of 0.1 MHz–0.1 Hz and a voltage amplitude of 5 mV. The effect of the binder in the cathode was measured using the galvanostatic intermittent titration technique (GITT) at 0.1 C. The battery discharged the pulse and rested for 2 h until the potential dropped to 2.8 V repeatedly. In the direct-current internal resistance test (DC-IR), the battery was first charged and discharged at a rate of 0.05 C. After charging to 4.5 V and standing for 1 h, the current rate was gradually increased (0.025 C–1 C). During the process, the battery was charged for 10 s, rested for 20 min, discharged for 10 s, and rested for another 20 min, and this process was repeated.

## 3. Results and Discussion

**Figure 2:**
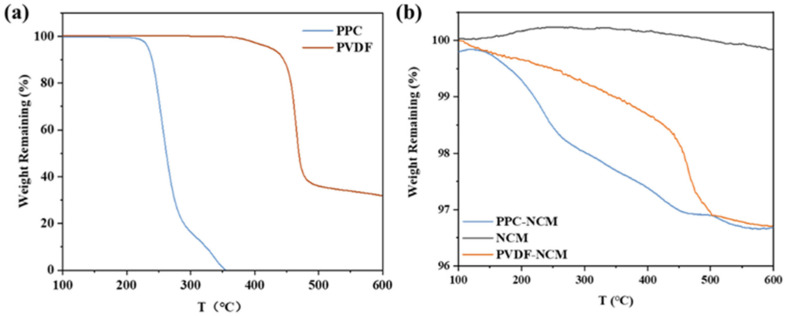
TGA curves of (**a**) PPC and PVDF binder and (**b**) PPC-NCM, PVDF-NCM cathode, and NCM811 powder.

TGA was used to investigate the thermal stability of the binder and its dispersion in the NCM811 cathode. In the temperature range of 100–600 °C, the binder and the corresponding cathode powder were randomly sampled for the TGA test. If the mass attenuation of the powder is about 2 wt.%, equal to the mass fraction of the added binder, it can be considered that the binder is uniformly distributed in the cathode powder. As shown in Figure 2a, the mass decay of PPC reaches 100% at 350 °C, so the PPC binder is easy to remove and the active materials and conductive additive can be effectively recovered. In previous studies, our group fully studied the degradation mechanism and thermal decomposition behavior of PPC and confirmed that PPC can be 100% decomposed by heat treatment [22,23]. It is noteworthy that both of them do not decompose at 170 °C, which suggests that the PPC binder can be adapted to the hot-pressing process in the solvent-free cathode preparation. Strong adhesion of the cathode material to the collector is crucial when manufacturing battery cathodes. To fulfill this requirement, it is necessary to ensure that the active material, conductive additives, and binder are uniformly distributed, so TGA tests were used to assess the homogeneity of the powder. Randomly selected mixed powders were tested. As shown in Figure 2b, the NCM811 powder did not show any mass degradation within 600 °C, while the PPC-NCM hybrid powder showed a mass degradation close to 2% at 350 °C, which is similar to the binder content we set. It can be assumed that the binder was uniformly distributed in the cathode.

**Figure 3:**
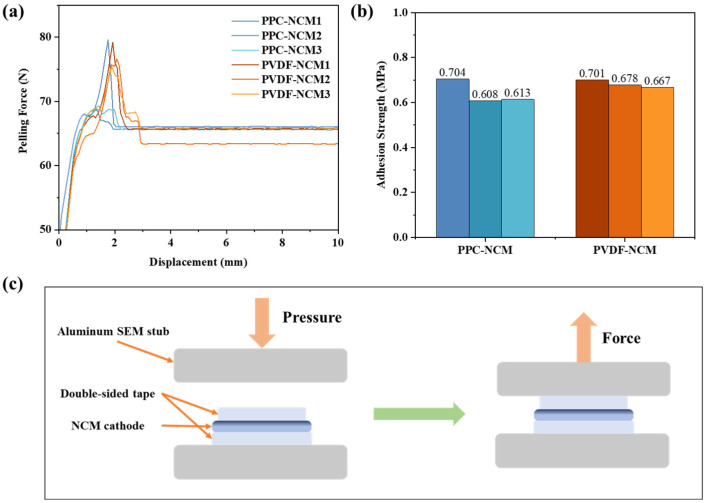
(**a**) Force–displacement curves during the peeling process of cathodes; (**b**) max peeling force of different cathodes; (**c**) schematic diagram of the cathode pull-off test.

Adhesion performance is one of the most important functions of battery binders. For the test and calculation of adhesion force, refer to the previous test methods [20,24]. The detailed method can be found in the Characterizations section. Figure 3a,b show the pull-off test results of the different cathodes, and the complete test procedure with a schematic diagram of the cathodes and tape assembly is shown in Figure 3c. The results show that the repeatability of adhesion in parallel tests is good and PPC has similar adhesion strengths with PVDF.

**Figure 4:**
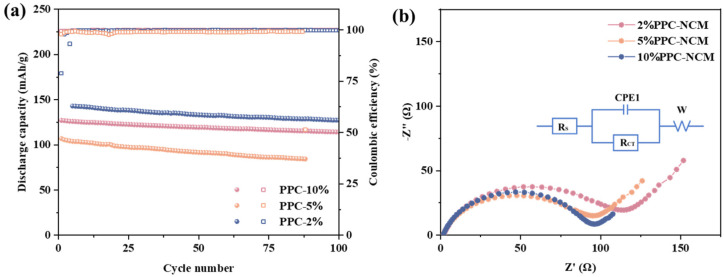
(**a**) Cycle performance and (**b**) EIS spectra of PPC-NCM cathode with different binder ratios.

The effect of different binder contents on lithium-ion batteries was first explored (Figure 4). PPC-NCM811 cathodes were prepared for charge/discharge tests according to different binder ratios, respectively. It was found that although the binder only occupied a small part in the cathode, the increase in PPC binder content caused a relatively obvious effect on the cycling performance of the battery.

As shown in Figure 4b, all cathodes show a similarly depressed semicircle, which corresponds to charge transfer resistance (Rct). After increasing the binder content, the impedance of the battery is slightly reduced. The Rct of the 10%PPC-NCM cathode is lower than that of the 2% PPC-NCM cathode, which is presumed to be due to the fact that the carbonate groups contained in PPC can help the adsorption and resolution of lithium ions, which is beneficial to the migration of lithium ions. Further research and experimentation could explore different combinations and concentrations of the binder to keep ion migration within the cathode materials, ultimately leading to advancements in battery technology. When the content of the PPC binder is 2 wt.%, the cycle is more stable, and the discharge capacity is significantly higher than that of other groups. Combined with the integrity and electrochemical performance of the prepared cathodes, subsequent experiments were prepared and tested at a binder content of 2 wt.%.

**Figure 5:**
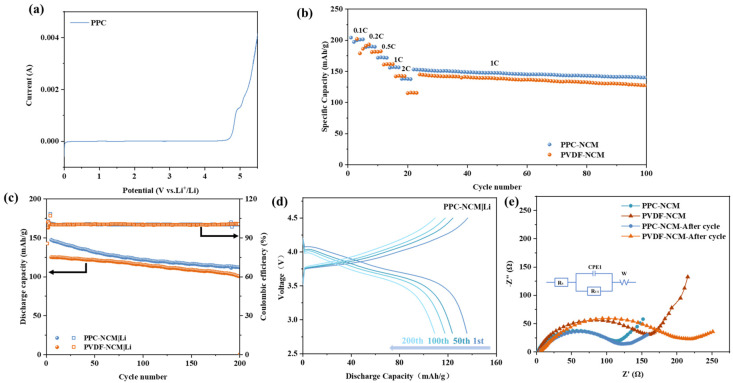
(**a**) Linear sweep voltammogram of PPC binder [25]; (**b**) rate performance of different cathodes at various current densities (between 0.1, 0.2, 0.5, 1.0, and 2.0 C); (**c**) the long-term cycle performance of different cathodes at 1 C; (**d**) the1st/50th/100th/200th charge/discharge curves of different cathodes at 0.5 C; (**e**) impedance changes in different electrodes before and after 100 cycles at 1 C.

In our previous work [25], we investigated the effect of PPC as a binder on the battery and the electrochemical stability of the battery, and LSV tests were performed. From the LSV test in Figure 5a, the electrochemical stability window of PPC is 0–5 V, indicating that the PPC binder will not have a redox reaction at the NCM cathode working voltage, proving its electrochemical stability, similar to the results of tests in previous studies [21]. Figure 5b shows the rate performance of PPC-NCM and PVDF-NCM cathodes. The capacities of the PPC-NCM cathode were 204.3 mAh/g, 187.7 mAh/g, 171.7 mAh/g, 156.2 mAh/g, and 137.7 mAh/g at different current densities from 0.1 C to 2 C. Compared with Hong’s [26] and Kang [27] et al.’s work, the rate performance has certain advantages. The discharge capacity of the PVDF-NCM cathode at all current densities was significantly lower than that of the PPC-NCM cathode, and it was only 115.9 mAh/g at 2 C. When the current density was back to 1 C, the capacities of both were restored to the initial level, and there was no obvious capacity degradation of PPC-NCM811 during the subsequent cycling process, indicating that the cathode prepared by PPC as a binder had good cycling stability.

The cycling performance of PPC-NCM and PVDF-NCM at room temperature is shown in Figure 5c. The initial discharge capacity of PPC-NCM at 1 C is as high as 147.5 mAh/g, and it still maintains a discharge capacity of 112.2 mAh/g with a coulombic efficiency close to 100% after 200 cycles. By contrast, the initial discharge capacity of PVDF-NCM is only 125.3 mAh/g, which is probably due to the poor conductivity and weak adhesion of PVDF. This makes PVDF unsuitable as a binder for high-load-capacity cathodes. Figure 5d shows the charging and discharging curves of PPC-NCM and PVDF-NCM cathodes at the 1st, 50th, 100th, and 200th cycles. The capacity stability of PPC-NCM is superior to that of PVDF-NCM cells. This is due to the fact that the PPC binder has a lower electrochemical impedance, which results in a lower polarization and higher stable discharge capacity of PPC-NCM during cycling. The impedance spectra of the PPC-NCM and PVDF-NCM cathode before and after cycling are illustrated in Figure 5e. The *X*-axis intercept corresponds to the internal resistance of the cathode, and the PPC-NCM cathode exhibits a lower interfacial resistance before cycling. After 100 cycles, the impedance of both PPC-NCM and PVDF-NCM cathodes increases; however, the impedance of PPC-NCM is still significantly smaller than that of PVDF-NCM. All cathodes exhibit a similarly depressed semicircle, corresponding to the charge transfer resistance (Rct). The Rct of the PPC-NCM cathode is lower than that of the PVDF-NCM cathode, presumably due to the beneficial effect of carbonate groups contained in PPC on the migration of lithium ions. Additionally, it is worth noting that this finding suggests potential for further optimization and development of cathode materials with improved performance for lithium-ion batteries. It shows that the conductive ability of the PPC binder can alleviate the increase in polarization to a certain extent, which can corroborate the previous experimental results, and it can be assumed that the PPC binder may help to maintain a good interfacial contact between the active material and the conductive additive during the cycling process.

**Figure 6:**
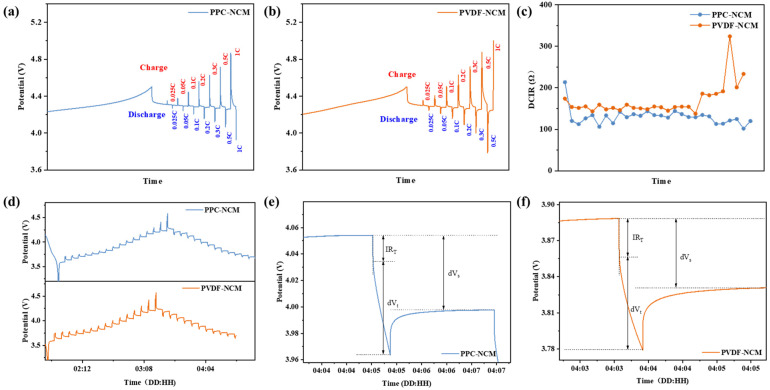
(**a**–**c**) Potential responses of (**a**) PVDF-NCM and (**b**) PPC-NCM. (**c**) Linear regression analysis. (**d**–**f**) GITT curves of PVDF-NCM and PPC-NCM.

DC-IR tests show the resistance of the cathodes prepared with two different binders and the voltage responses of the two cells, as shown in Figure 6a–c, respectively. As the current density increases from C/40 to 1 C, the polarization of PPC-NCM is smaller than that of PVDF-NCM, indicating that PPC has better ionic conductivity. Linear regression analysis shows that the resistance of PPC-NCM is smaller than that of PVDF-NCM during both cycling. GITT was additionally used to evaluate the effect of the binder on the cathode (Figure 6d–f). The lithium-ion diffusion coefficient D can be calculated from Equation (1), and the diffusion coefficient will determine the reaction rate, which affects the performance of the battery, where D is the lithium-ion diffusion coefficient, τ is the relaxation time, n_m_ is the mole number, V_m_ is the molar volume of the electrode material, and S is the contact area between the electrode and the electrolyte, so a quantitative comparison of the lithium-ion coefficients can be realized by comparing the magnitude of (ΔE_S_)/(ΔE_t_).

(1) $$D=\frac{4}{\pi \tau }({\frac{n_{m}V_{m}}{S})}^{2}({\frac{\Delta E_{s}}{\Delta E_{t}})}^{2}$$

As the cycling process is repeated, the cell polarization gradually increases. The lithium-ion diffusion coefficients of PVDF-NCM and PPC-NCM cells are quantitatively compared around 70 h. For PVDF-NCM cells, (ΔE_S_)/(ΔE_t_) is 0.74. For PPC-NCM cells, (ΔE_S_)/(ΔE_t_) is 0.81. As the cycling proceeds, the PPC-NCM cells show lower polarization, while the PVDF-NCM cells quickly exhibit larger polarization. The above test results indicate that PPC stabilizes the lithium-ion battery interface through its high ionic conductivity and uniform dispersion of cathode materials, improves lithium-ion transport efficiency, and reduces the degree of increase in battery impedance and polarization during cycling.

**Figure 7:**
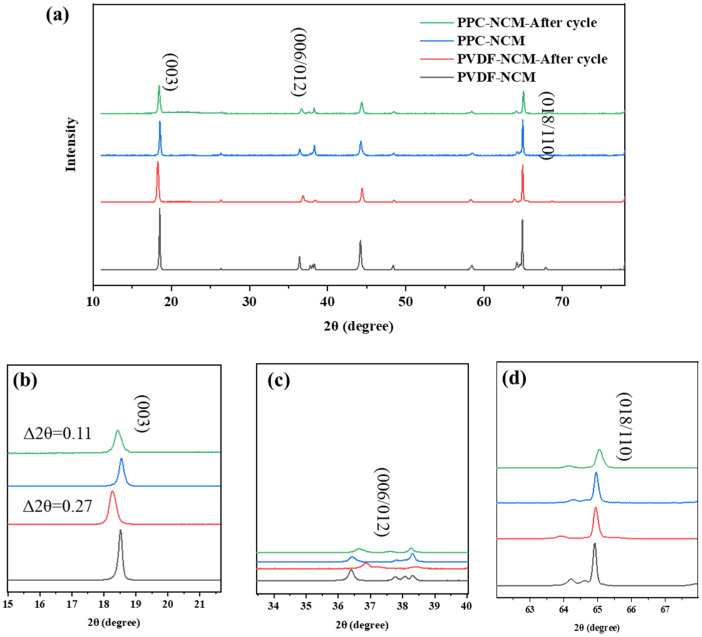
XRD curves of (**a**) PPC-NCM and PVDF-NCM before and after cycling. Corresponding to the partial magnification of diffraction peaks of (**b**) (003), (**c**) (006)/(102), and (**d**) (018)/(110).

In order to further analyze the relationship between the electrode structure and its electrochemical performance, XRD tests were performed on cathodes before and after cycling (Figure 7a–d). The NCM material exhibits a phase transition from a layered structure to a spinel–rocksalt phase during cycling. There are sharp diffraction peaks of (003), (006)/(102), and (018)/(110), referring to a layered structure. The degree of bimodal splitting of (006/102) and (018/110) in the spectra is the key basis for judging the layered structure of NCM811 material [28,29,30]. It was found that the degree of their bimodal splitting changed before and after the cycling of both electrodes, but the changes in the PPC-NCM cathode was smaller, which indicated that the degree of irreversible phase transition was smaller. After cycling, magnifying the partial XRD patterns, peak 003 shifted to the left to different degrees, particularly for the PVDF-NCM cathode, indicating that the lattice volume was changed. However, the degree of left shift of the PPC-NCM cathode, which is only 0.11°, is smaller (compared with 0.27° of the PVDF-NCM cathode), indicating that its lattice volume changes are small. The splits of the (006)/(102) doublet of the PVDF-NCM cathode become weaker or even disappear as compared to the PPC-NCM cathode. The intensity of the peak (018)/(110) also indicates that PPC binders possess the capability to stabilize the cathode structure. The above results indicate that the PPC binder can increase the cycle life of the battery by better protecting the layer structure of the cathode material.

**Figure 8:**
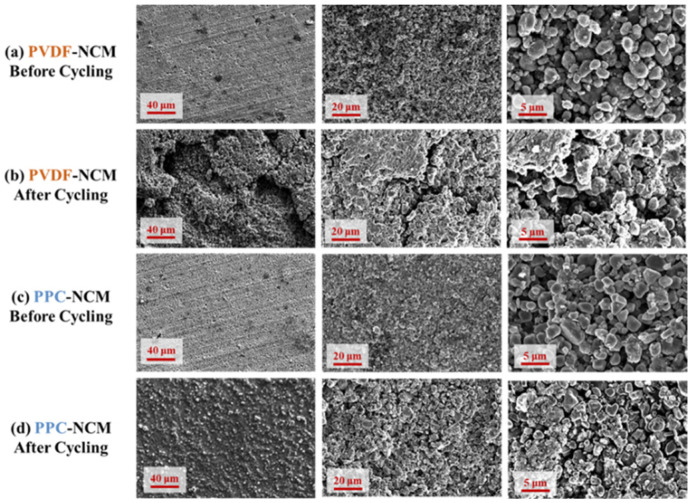
SEM images of different electrodes (**a**,**c**) before and (**b**,**d**) after 200 cycles.

**Figure 9:**
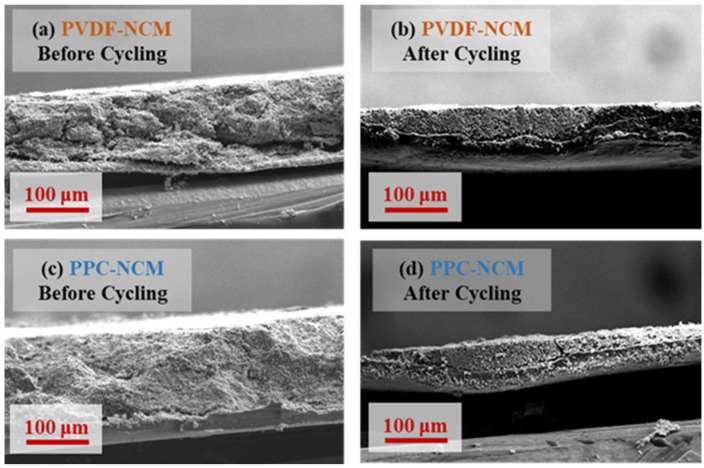
SEM images of (**a**,**b**) PVDF-NCM and (**c**,**d**) PPC-NCM cross-sections before and after cycling.

The SEM images of PPC-NCM and PVDF-NCM before and after cycling are shown in Figure 8. At low magnification, the surface morphology of PVDF-NCM and PPC-NCM cathodes before cycling is similar. When studying more detailed morphologies and particles, the PVDF-NCM cathode shows particle stripping and shedding. In contrast, the PPC-NCM cathode has uniformly dispersed conductive carbon black around the NCM811 particles and forms a tight conductive network, showing that the PPC binder has good dispersion and adhesion. After 200 cycles, the SEM images of the PPC-NCM cathode show a more intact surface without material stripping, indicating that PPC as a binder improves the stability of the cathode structure. The PVDF-NCM cathode material shows cracks after the long-cycle test, indicating that it has poor adhesion and is unable to endure the stresses caused by the volumetric changes in the cycling, which may lead to the cathode material being partially or completely stripped off from the collector, which may reduce the cycling capacity. In Figure 9, we prepare and compare the cross-sections of PVDF-NCM and PPC-NCM cathodes before and after cycling under the same conditions. Compared with PVDF-NCM, PPC-NCM is denser and does not show cracks throughout the interface. After cycling, the thickness of the cathode is reduced, possibly due to the residual part of the wetted and shed electrode powder on the diaphragm. The cross-section of PPC-NCM is more complete, but PVDF-NCM shows a clear crack between the collector and the cathode powder, which is consistent with the state before cycling and can also explain the attenuation of capacity.

**Figure 10:**
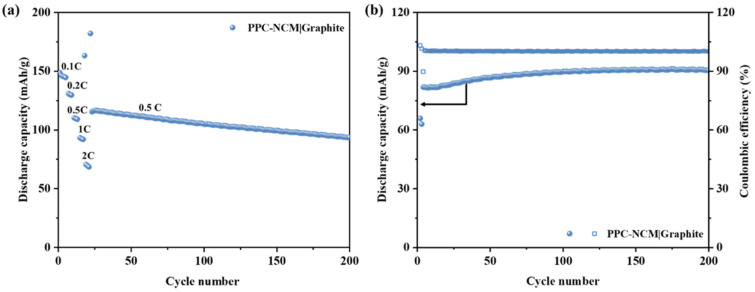
(**a**) Rate performance and (**b**) cycle performance of PPC-NCM|graphite full cell at 0.5 C.

In order to explore the potential of the binder and process for commercial application, batteries with commercially available graphite as the anode were assembled and characterized electrochemically (Figure 10). It was found to have a stable cycling at 0.5 C with high-capacity retention. The specific capacity was 148.6 mAh/g at a current density of 0.1 C during rate performance tests. With the increase in current density, the discharge capacity decreased. When the current density returned to 0.5 C, the discharge capacity returned to 115.5 mAh/g and stabilized the cycle to 200 cycles. This indicates that the NCM cathode prepared by the solvent-free method with PPC as the binder has potential for commercial application.

## 4. Conclusions

In this work, a simple solvent-free preparation method for lithium-ion battery cathodes is presented, in which the cathode can be obtained by high-speed stirring and the hot-pressing process without the need to introduce solvents, dispersants, or other additives. The PPC binder has a conductive ability and a wide electrochemical stabilization window suitable for lower-temperature production processes. Especially after the battery fails, the binder can disappear 100% at a temperature that is not too high, which is extremely convenient for the recycling of electrode materials. So, this work provides a new idea and method for a more efficient, fast, and environmentally friendly cathode fabrication for lithium-ion batteries. Solvent-free cathodes with PPC and PVDF as binders were prepared under the same preparation conditions, and both of them showed excellent thermal stability and could be stabilized during cathode preparation and battery fabrication. The PPC-NCM cells showed smaller polarization and more stable cathode morphology characteristics by DCIR, GITT, and other tests. The constitutive relationship between the PPC binder and NCM cathode was investigated using XRD and SEM tests, and it was found that the PPC binder could effectively inhibit the electrode surface side-reactions and stabilize the electrode structure. PPC-NCM has excellent cycling stability and rate performance, which can be stably cycled for 200 cycles at 1 C. The discharge capacity after 200 cycles is 112.2 mAh/g, and the capacity retention rate is 76.1%. The cycling performance is significantly better than that of PVDF-NCM. Finally, the PPC-NCM/graphite full cell verifies that this work has potential for practical applications.

## Data Availability

Data are available from the authors upon reasonable request.
